# Severe skin toxicity and early progression following neoadjuvant ensartinib and surgery in anaplastic lymphoma kinase-positive locally advanced lung cancer: a case report 

**DOI:** 10.3389/fphar.2025.1673086

**Published:** 2025-09-18

**Authors:** Hongming Wang, Shiyan Li, Zhijun Wu, Wei Xu, Nuoni Wang, Zemin Xiao

**Affiliations:** ^1^ Department of Oncology, Changde Hospital, Xiangya School of Medicine, Central South University, The First People’s Hospital of Changde City, Changde, China; ^2^ Department of Thoracic Surgery, Changde Hospital, Xiangya School of Medicine, Central South University, The First People’s Hospital of Changde City, Changde, China; ^3^ Department of Electrophysiology, Changde Hospital, Xiangya School of Medicine, Central South University, The First People’s Hospital of Changde City, Changde, China

**Keywords:** neoadjuvant ensartinib, anaplastic lymphoma kinase (ALK), lung cancer, skin toxicity, early progression

## Abstract

**Background:**

Anaplastic lymphoma kinase (ALK) fusion mutations exhibit exceptional sensitivity to tyrosine kinase inhibitors (TKIs) in patients with advanced non-small cell lung cancer (NSCLC). Ensartinib, a second-generation ALK-TKI, represents a promising therapeutic option for ALK mutation-associated NSCLC; however, its clinical application in perioperative therapy remains to be elucidated.

**Case description:**

We report the case of a 45-year-old female diagnosed with stage IIIA (cT2N2M0, AJCC eighth edition) adenocarcinoma of the right lung harboring an EML4-ALK fusion (E6:A20) and a TP53 mutation. Following 3-month neoadjuvant therapy with ensartinib, surgical conversion from R(un) to R0 resection was achieved, accompanied by histopathological assessment and confirmation of a major pathological response (MPR) (<10% viable tumor cells) and negative postoperative molecular residual disease (MRD) surveillance. Despite effective neoadjuvant targeted therapy and the absence of significant adverse events, the patient experienced drug-refractory grade 3 cutaneous toxicity (CTCAE v5.0) 4 weeks after surgery and was subsequently found to have a T12 vertebral metastasis on 3-month surveillance imaging. After multidisciplinary evaluation and considering the patient’s refusal to undergo local therapies, treatment was switched to lorlatinib. The patient subsequently experienced complete resolution of skin toxicity, sustained disease control, and a significantly improved quality of life.

**Conclusion:**

This case report describes a patient with an MPR subsequent to neoadjuvant ensartinib, who nonetheless developed early postoperative progression. Our case cautions that although MPR and MRD negativity can strongly predict lower recurrence risk, these markers may not universally guarantee long-term remission in every individual. The case underscores the need for continued vigilance and individualized surveillance strategies even once favorable pathological responses are achieved. Additionally, the perioperative evolution of skin toxicity highlights the importance of continuous adverse event monitoring and management.

## 1 Introduction

Non-small cell lung cancer (NSCLC) represents approximately 85% of all diagnosed lung cancer cases, with adenocarcinoma being the predominant subtype, followed by squamous cell carcinoma (Thai et al., 2021). With the development of molecular detection technology, lung cancer driver genes such as epidermal growth factor receptor (EGFR) variants and anaplastic lymphoma kinase (ALK) fusions have been identified. Targeted therapeutic agents directed against these specific alterations have demonstrated superior efficacy and safety compared to traditional chemotherapy (Tan and Tan, 2022). The most common ALK fusion gene in patients with NSCLC is EML4-ALK, with an incidence of 3%–7% (Gandhi et al., 2015). More than 20 EML4-ALK variant subtypes have been identified, with variants 1 and 3 collectively accounting for over 60% of cases (Molecular Pathology Collaboration Group of Tumor Pathology Committee of China Anti-Cancer Association et al., 2023). Whether ALK-tyrosine kinase inhibitor (TKI) efficacy differs among patients harboring distinct EML4-ALK variants remains inconclusive (Zhang et al., 2021). Several ALK-TKIs are clinically available, all demonstrating favorable efficacy with manageable toxicity, and have been approved for treating advanced ALK-mutant NSCLC. Among these, treatment with ensartinib, a second-generation ALK-TKI developed in China, achieved a median progression-free survival (PFS) exceeding 25 months in the eXalt3 trial (NCT02767804) while maintaining a favorable safety profile (11.2% incidence of grade 3 rash) (Horn et al., 2021).

Stage III NSCLC exhibits significant heterogeneity, posing a clinical challenge for optimal treatment selection. Unlike stage IV disease, stage III NSCLC is potentially curable and warrants aggressive management. Multidisciplinary team consensus is essential for defining individualized treatment, and requires input from thoracic surgery, radiation oncology, medical oncology, respiratory medicine, pathology, and radiology specialists. For patients harboring driver gene-negative stage II–III NSCLC, neoadjuvant chemotherapy has been replaced by neoadjuvant immunotherapy combined with chemotherapy, unless contraindications to immunotherapy exist. This paradigm shift is supported by robust evidence from multiple large-scale phase III clinical trials (Forde et al., 2022; Wakelee et al., 2023; Yue et al., 2025). For patients with stage II–III NSCLC harboring EGFR mutations or ALK rearrangements, significant progress has been made in the adjuvant setting. Although several targeted agents, including osimertinib, alectinib, and icotinib, have received regulatory approval in the neoadjuvant landscape, no targeted therapy has yet gained formal approval. Nevertheless, multiple clinical trials are actively underway (Lee et al., 2023). We anticipate more high-quality clinical trial results that will provide optimized treatment options for patients with driver mutation-positive locally advanced NSCLC.

This report describes the case of a patient with stage IIIA ALK-positive NSCLC who underwent R0 resection following neoadjuvant therapy with ensartinib. The patient achieved a major pathological response (MPR) and pathological downstaging but developed a grade 3 rash and experienced early postoperative progression, highlighting the multifaceted challenges in the perioperative use of ALK-TKIs.

## 2 Case presentation

### 2.1 Clinical information

**FIGURE 1:**
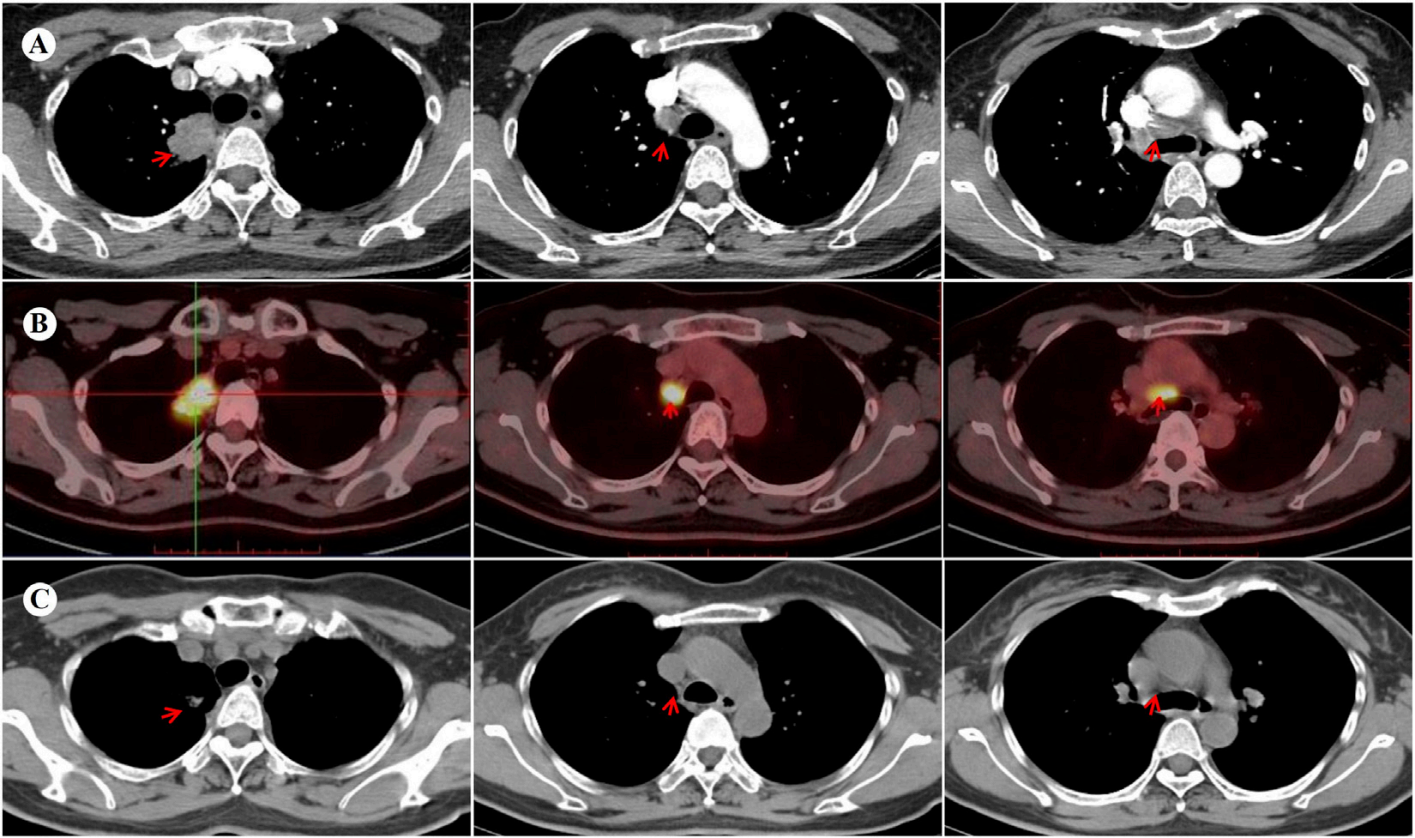
Images before and after neoadjuvant targeted therapy with ensartinib. **(A)** Contrast-enhanced chest CT at baseline demonstrates a 32 × 24 mm mass with heterogeneous enhancement in the right upper lobe, accompanied by enlarged mediastinal lymph nodes (stations 2R/4R). **(B)** The baseline PET-CT performed on 21 November 2023 reveals hypermetabolism in the primary lesion (maximum standardized uptake volume [SUVmax] 14.8) and mediastinal lymph nodes (SUVmax 15.5), without distant metastasis. **(C)** Follow-up lung CT at 3 months post-neoadjuvant therapy shows a significant reduction of the primary lesion (11 × 7 mm) and mediastinal lymph nodes, indicating a partial response (PR) to ensartinib. CT, computed tomography; PET, positron emission tomography.

A 45-year-old Chinese woman incidentally presented with a 32 × 24 mm mass in the right-upper lung lobe with enlarged mediastinal nodes (stations 2R and 4R per IASLC classification) on chest computed tomography (CT) images during a checkup visit on 14 November 2023. The findings were suggestive of lung cancer with possible lymph node metastases (Figure 1A). The patient was asymptomatic at presentation, with unremarkable physical examination and an Eastern Cooperative Oncology Group (ECOG) performance status of 0. The medical history was notable for being negative regarding active/passive smoking, excessive alcohol consumption, and occupational carcinogen exposure. Family history was unremarkable for hereditary cancer syndromes or genetic disorders, with no documented malignancies in first-degree relatives across three generations. Bronchoscopy was unremarkable; however, CT-guided transthoracic needle biopsy of the pulmonary lesion confirmed invasive adenocarcinoma. Staging magnetic resonance imaging (MRI) of the brain and positron emission tomography (PET)-CT (Figure 1B) confirmed cT2N2M0 (AJCC, 8th edition) disease. Next-generation sequencing (NGS) identified an EML4-ALK fusion (E6:A20) and TP53 mutation with programmed death-ligand 1 expression corresponding to a tumor proportion score of 1%.

### 2.2 Course of treatment

Given the metastatic involvement of upper mediastinal lymph nodes (station 2R), the surgical outcome was initially classified as an uncertain resection [R(un)]. Following multidisciplinary consensus, neoadjuvant ensartinib 225 mg daily was initiated on 11 December 2023. No significant adverse effects were observed. Imaging after 3 months revealed a partial response (PR) per RECIST 1.1 criteria, with significant regression of the primary lesion and metastatic lymph nodes (Figure 1C). Subsequently, the patient underwent a right-upper lung lobectomy with systematic lymph node dissection on 15 March 2024. Postoperative pathology confirmed an MPR in the primary lesion, with <10% viable tumor cells and accompanied by abundant foam cells and lymphocytes. Notably, the resected lymph nodes demonstrated complete pathological clearance of malignant cells. Final pathological staging was designated as pT1bN0M0 according to the AJCC eighth edition criteria. Adjuvant ensartinib maintenance was recommended.

### 2.3 Toxicity and disease progression

**FIGURE 2:**
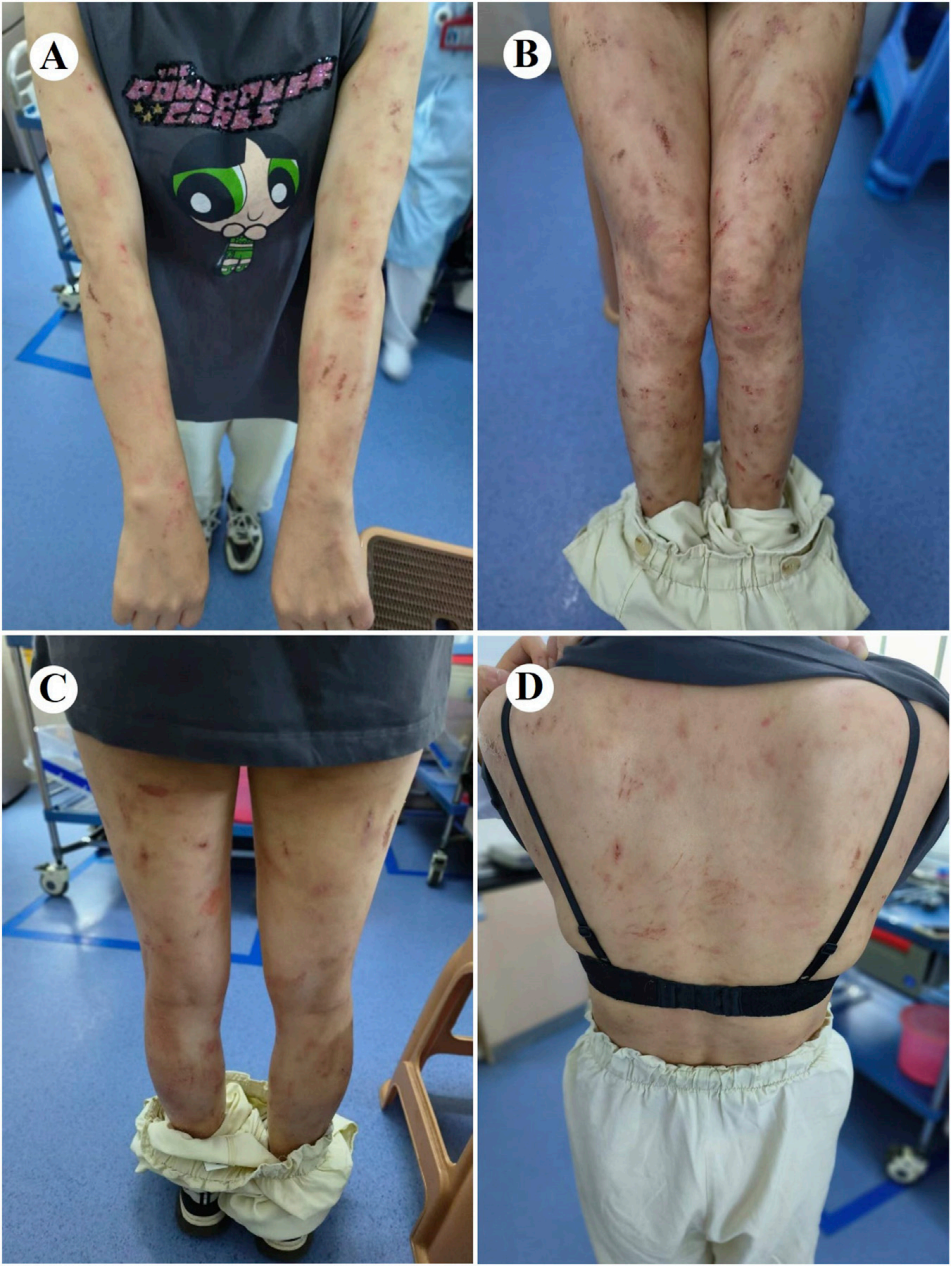
Cutaneous adverse events associated with ensartinib therapy. Grade 3 skin toxicity developed postoperatively in this patient, characterized by rash, pruritus, and visible scratching on the extremities **(A–C)** and trunk **(D)**, consistent with CTCAE v5.0 criteria.

**FIGURE 3:**
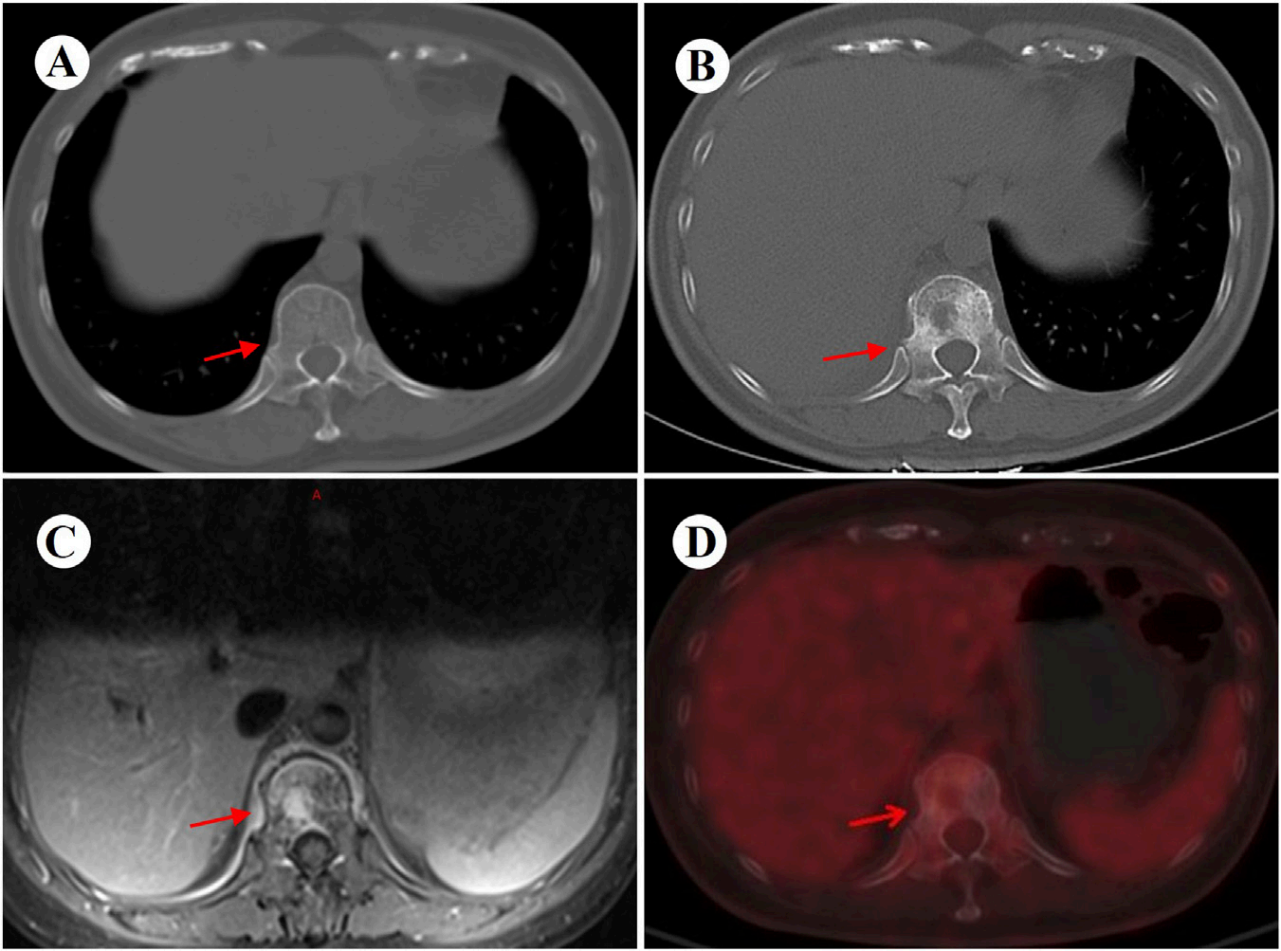
Images before and 3 months after surgery. **(A)** Preoperative chest CT (bone window) performed on 13 March 2024 shows an intact T12 vertebral body and attachments. **(B)** Follow-up CT (bone window) at 3 months postoperatively reveals new osteolytic destruction of the T12 vertebral body and attachments. **(C,D)** Subsequent MRI and PET-CT further confirm metastatic involvement of the T12 vertebra, with concordant imaging findings. CT, computed tomography; PET, positron emission tomography; MRI, magnetic resonance imaging.

At 4 weeks postoperatively, the patient developed CTCAE v5.0 grade 3 dermatological toxicity characterized by a pruritic rash. This adverse event persisted despite stepwise dose reduction (from 225 mg to 150 mg) and combination therapy with systemic corticosteroids and antihistamines (Figure 2). Notably, serial molecular residual disease (MRD) assessments were negative at 10 days and 3 months postoperatively. However, surveillance imaging at the 3-month follow-up demonstrated osteolytic destruction of the T12 vertebral body and adjacent structures, radiologically consistent with osseous metastasis (Figure 3). Re-staging indicated rT0N0M1 (Stage IV) disease. For oligometastatic bone disease, local surgery or radiotherapy may be considered alongside ongoing targeted therapy. The patient, however, declined further local interventions, including radiotherapy or surgical management, citing diminished confidence following early disease progression.

### 2.4 Adjustment of treatment program and follow-up

**FIGURE 4:**
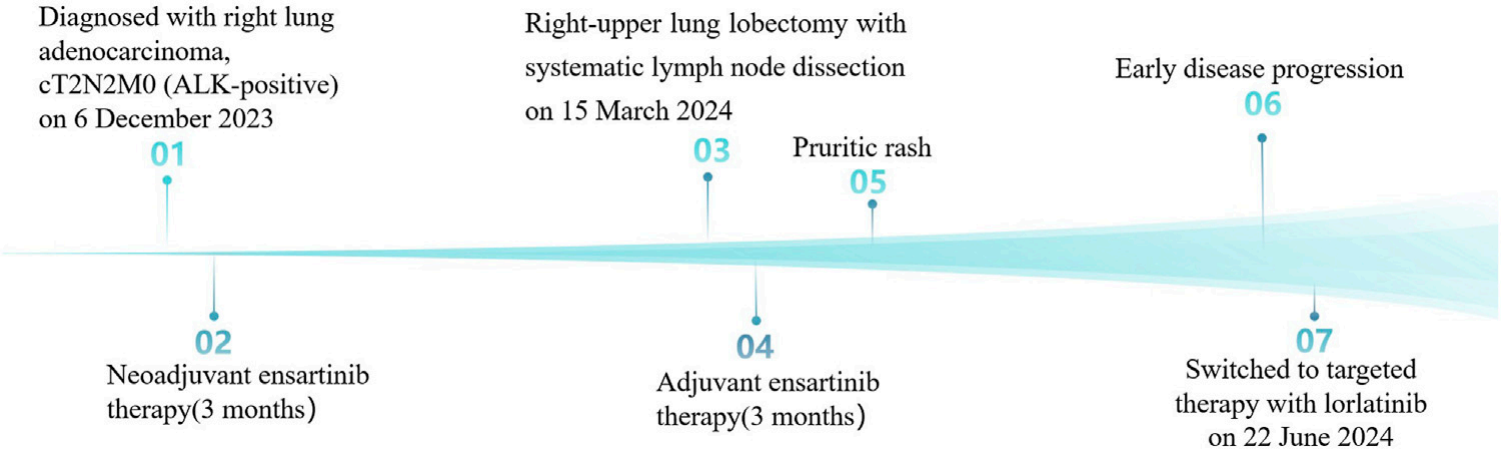
The patient’s therapeutic timeline.

Following reassessment by the multidisciplinary team, ensartinib was switched to lorlatinib (100 mg daily) on 22 June 2024, which achieved complete resolution of cutaneous toxicity within 14 days, although asymptomatic grade 1 hyperlipidemia (CTCAE v5.0) remained, requiring atorvastatin management. Serial radiographic surveillance demonstrated sustained disease stability with preserved quality of life (ECOG PS 0) through the 13-month follow-up. Longer-term monitoring will evaluate response durability and inform future clinical strategies. Her therapeutic timeline is depicted in Figure 4.

## 3 Discussion

NSCLC represents a paradigm of precision therapy. ALK fusion-targeted treatment significantly benefits patients and has earned the “diamond target” designation. However, not all ALK fusion variants respond well to ALK-TKIs, as they rarely occur alone and often co-mutate with other genes. These variants and co-mutations complicate clinical management (Zhang et al., 2021). The variant v3a/b (E6a/b: A20), one of the most common EML4-ALK variants, confers a worse prognosis; patients experience earlier treatment failure and shorter overall survival (OS) (Christopoulos et al., 2018). Although the prognostic significance of TP53 mutations (TP53mt) remains debated, the more frequently co-occurring genomic alterations in ALK-rearranged NSCLC universally correlate with reduced PFS and OS following ALK TKI therapy (Zhang et al., 2021).

In a real-world study that included 307 patients with advanced ALK-positive NSCLC, 50% of patients harbored the ALK fusion V1, and 36% had ALK fusion V3. The co-occurrence frequencies of ALK fusion V1 with TP53mt and ALK fusion V3 with TP53mt were 18% and 14%, respectively. For the entire cohort, the median time to discontinuation (TTD) for first-line targeted therapy was 19.9 months (95% confidence interval [CI]: 13.8–28.7). By subgroup analysis, the median TTD was not evaluable (NE) (95% CI: 14.3–NE) for patients with TP53-ND (TP53 mutation not detected)/V1, compared with 17.1 months (95% CI: 8.7–37.5) for patients with TP53mt/V1. For patients harboring the TP53-ND/V3 variant, the median TTD was 23.8 months (95% CI: 9.4–NE), compared with only 7.4 months (95% CI: 4.2–31.1) for patients having the TP53mt/V3 variant (Parikh et al., 2024). Our case also demonstrates the poor prognosis of the ALK-TP53mt/V3 variant, indicated by a TTD of approximately 6 months, which is consistent with the result of the real-world study described above.

The EML4-ALK variants most described in clinical studies are variant 1 (V1) and variant 3a/b (V3) (Zhang et al., 2021). Designated a “short” variant, V3 lacks the hydrophobic EMAP-like protein (HELP) motif within the tandem atypical propeller (TAPE) domain compared with the V1 variant (Bayliss et al., 2016; Heuckmann et al., 2012). An *in vitro* study demonstrated that fusion protein stability correlated with reduced sensitivity to ALK inhibitors (Heuckmann et al., 2012). The absence of the HELP domain confers greater protein stability to EML4-ALK V3. This variant is associated with a higher incidence of the solvent-front mutation G1202R, which impairs drug binding to first- and second-generation ALK inhibitors (Lin et al., 2018). Alternative resistance mechanisms linked to V3 include non-EML4-ALK fusions and increased expression of the EML4-ALK RNA isoform, both of which are associated with worse survival (Song et al., 2022; Zhou et al., 2022). Furthermore, patients harboring variant 3 demonstrated higher tumor mutational burdens (TMBs) (Ou et al., 2017).

TP53 mutations correlate with increased chromosomal instability and MYC amplification (Alidousty et al., 2018). Greater chromosomal instability itself predicts worse PFS (Zheng et al., 2020). Furthermore, TP53 mutations are clinically categorized as disruptive or nondisruptive, and the latter correlate with worse OS (Canale et al., 2022). The oncogenic features of TP53 mutations and the V3 variant may act independently or exhibit additive/synergistic effects. Although uncommon, the dual-positive status (TP53mut/V3 variant) signifies a markedly poor prognosis. This likely stems from the combined impact of V3’s propensity for rapid acquisition of drug-resistant mutations and TP53-driven genomic instability. Together, these features facilitate tumor cell adaptation through alternative pathway activation and drive accelerated tumor evolution (Chan et al., 2025).

Neoadjuvant targeted therapy for locally advanced NSCLC is an innovative strategy, aiming to reduce tumor size, improve surgical resectability, and thereby enhance postoperative survival rates. Currently, several clinical trials evaluating neoadjuvant targeted therapies have shown preliminary positive results that warrant further research and exploration (Lee et al., 2023). The NEOEAST study (NCT05380024) is a single-arm, open-label, prospective, phase II study with the objective of assessing the feasibility of ensartinib as neoadjuvant therapy in patients with resectable stage II-IIIB ALK fusion-positive NSCLC. The primary endpoint is MPR. The study started in May 2022 and is currently ongoing (Lu et al., 2023). At the ASCO 2025 meeting, real-world evidence from a retrospective study of adjuvant ensartinib in ALK-positive NSCLC reported a 2-year DFS rate of 92.1% across the cohort. Although OS data remain immature, ensartinib demonstrated encouraging efficacy with a favorable safety profile in resectable ALK-positive NSCLC (Wang et al., 2025). A critical Phase III randomized controlled trial (NCT05341583) is currently evaluating the efficacy and safety of ensartinib as adjuvant therapy in patients with resectable ALK-positive NSCLC at stages IB-IIIB. This study has now completed enrollment and is expected to address the gap in targeted adjuvant therapies for this patient population within the context of domestically developed drugs in China.

To date, three published case reports have described ensartinib as neoadjuvant therapy for locally advanced ALK-positive NSCLC. All patients achieved tumor downstaging with complete surgical resection, demonstrating favorable pathological responses: one attained a pathological complete response (pCR) and two others achieved an MPR. Adverse events included mild rash (*n* = 2) and grade 4 edema (*n* = 1), the latter resolving after dose reduction/diuretic therapy. Postoperative surveillance revealed no disease progression in any patient (Wang et al., 2022; Wu et al., 2023; Zhang et al., 2025). Although current evidence remains limited, ensartinib demonstrates promise as a neoadjuvant option for resectable ALK-positive NSCLC.

In our case, neoadjuvant targeted therapy with ensartinib was successful, the surgery achieved R0 resection, and postoperative histopathology showed an MPR, indicating that the tumor was highly sensitive to the treatment. Additionally, the postoperative molecular residual disease surveillance results were negative, suggesting a low risk of recurrence and a favorable prognosis. Nonetheless, 3 months after surgery, the patient developed bone metastasis, marking the failure of postoperative adjuvant therapy. Our results indicate that ALK mutation subtypes and co-mutations may be associated with therapeutic response and prognosis following neoadjuvant targeted therapy in early-stage NSCLC. Incorporating these genetic characteristics into regimen selection may be justified, pending confirmation in large-scale clinical studies. The early onset of bone metastases may indicate underlying tumor heterogeneity, potentially contributing to resistance to ensartinib. It is currently believed that the primary cause of postoperative recurrence in lung cancer is owing to the presence of preoperative occult micrometastases. However, these small metastases are beyond the detection capabilities of current imaging techniques. Over time, these hidden foci may “resurrect” and proliferate, eventually leading to tumor recurrence. Theoretically, since postoperative histopathology is not reflective of distant occult metastases, even patients evaluated as having achieved a pCR remain at risk for postoperative recurrence. Indeed, based on available data, approximately 15% of surgical patients who achieve a pCR after neoadjuvant immunochemotherapy will experience disease recurrence within 3 years (Cascone et al., 2024; Spicer et al., 2024).

Pathological evaluation of surgically resected lung cancer specimens after neoadjuvant therapy has become increasingly important with the growing use of pathological responses in clinical trials. Pathological response after neoadjuvant therapy serves as a validated prognostic indicator across multiple cancers, where a pCR and an MPR generally correlate with improved survival (Allen et al., 2021; Shen et al., 2021). A meta-analysis demonstrated that achieving MPR correlates with improved OS in patients with resectable NSCLC receiving neoadjuvant immunochemotherapy (Chen et al., 2023). Another study demonstrated that in patients with potentially resectable NSCLC receiving neoadjuvant immunochemotherapy, MPR and pCR were significantly associated with improvement in PFS; however, when the effects of pCR and MPR were directly compared, no significant differences were observed in their effects on PFS (hazard ratio: 0.20, 95% CI: 0.02–2.21, *P* = 0.15) (Xu et al., 2025). The evaluation of pathological response following neoadjuvant targeted therapy has emerged as a critical prognostic determinant in resectable NSCLC.

In perioperative NSCLC management, circulating tumor DNA-based MRD (ctDNA-MRD) monitoring has emerged as a promising method for postoperative surveillance. ctDNA-MRD identifies potentially curable populations (Zhang J. T. et al., 2022) and patients with a high recurrence risk (Abbosh et al., 2023), which provides important prognostic stratification. Beyond prognostic utility, ctDNA-MRD demonstrates predictive value for guiding adjuvant therapy decisions—including treatment escalation or de-escalation—following curative resection. An observational study demonstrated that postoperative ctDNA-negative patients had a lower risk of recurrence regardless of whether they received adjuvant chemotherapy or not, whereas postoperative ctDNA-positive patients benefited from postoperative adjuvant chemotherapy (Qiu et al., 2021). In a prospective study evaluating adjuvant osimertinib in patients with resectable EGFR-mutated NSCLC, circulating tumor DNA (ctDNA)-based MRD detection strongly predicted recurrence risk, with recurrence identified at a median lead time of 4.7 months. Notably, 25% of osimertinib-treated patients experienced recurrence despite sustained MRD-free status in the majority cohort. These findings underscore the value of MRD monitoring in identifying high-risk patients and personalizing treatment strategies. Furthermore, although only 8% of patients exhibited detectable MRD at randomization, this subgroup still derived significant clinical benefit from osimertinib, reinforcing the clinical utility of MRD assessment in treatment decision-making (Herbst et al., 2025). Ongoing phase III trials (e.g., the MERMAID-1 [NCT04385368], MERMAID-2 [NCT04642469] studies) are prospectively evaluating ctDNA-MRD-guided adjuvant strategies in NSCLC, which will provide critical evidence for MRD-directed postoperative management.

The most common adverse reactions to ensartinib were skin-related adverse reactions, consistent with the results of previous studies (Horn et al., 2021; Yang et al., 2020). The pathogenesis of ensartinib-induced cutaneous adverse events (e.g., rash) remains incompletely characterized. Preclinical evidence indicates ALK expression in normal epidermal tissue, where ALK tyrosine kinase inhibitors suppress human keratinocyte proliferation *in vitro* (Ning et al., 2013). The high skin permeability of ensartinib increases the risk of dermatological toxicity. Cutaneous adverse events require graded therapeutic interventions based on their severity. Most cutaneous adverse reactions were grades 1–2, were generally well tolerated, and typically followed a self-limiting course, resolving with symptomatic management. The median time to onset was 9 days, with a median duration of 21.5 days. Primary rash morphologies included maculopapular, macular, and papular eruptions, predominantly affecting the face, trunk, and extremities. Associated features included desquamation, pruritus, edema, and occasional fever (Zhang and Yang, 2022b). The development of a rash may be associated with superior clinical efficacy of ensartinib (Wakelee et al., 2019); however, in our case, the opposite was observed. The patient was treated with a full dose of preoperative ensartinib orally for 3 months, which was effective and well tolerated, with no significant skin toxicity or other adverse events. In contrast, after 4 weeks of continued postoperative adjuvant therapy with the same dose of ensartinib (225 mg/d), the patient gradually developed severe grade 3 skin toxicity. Despite dose reduction and symptomatic treatment, the patient did not experience relief from the skin toxicity, and early disease progression was noted. Surgical trauma triggers systemic stress and inflammatory responses, activating neuroendocrine pathways and cytokine cascades, particularly elevated IL-6 levels, thereby altering the immune ‘set point’ and potentially lowering the threshold for T cell-mediated hypersensitivity reactions and skin inflammation (Desborough, 2000; Tanaka et al., 2014). Concurrently, systemic inflammation and stress-related mediators can compromise epidermal barrier homeostasis and disrupt skin immune tolerance (Proksch et al., 2008). These mechanisms provide a pathophysiological basis for the observed accumulation of severe skin toxicity following treatment with ensartinib in the postoperative context. Although the rash appeared late in this patient, a drug-induced delayed cutaneous reaction cannot be definitively excluded. Further mechanistic studies and clinical trials are warranted to substantiate these observations.

In conclusion, the case described herein supports the use of ensartinib as a promising targeted agent for neoadjuvant treatment of locally advanced ALK-mutant NSCLC. Nonetheless, based on previous literature, the established biomarkers, MPR and MRD, demonstrate incomplete predictive value for therapeutic efficacy. It should be kept in mind that treatment outcomes are also modulated by specific mutant subtypes and co-occurring genomic alterations, necessitating a comprehensive molecular characterization. Furthermore, the hierarchical management of toxicities associated with perioperative targeted therapy is crucial for ensuring patient quality of life and successful outcomes.

## Data Availability

The original contributions presented in the study are included in the article/supplementary material, further inquiries can be directed to the corresponding authors.

## Patient perspective

The patient recognized our efforts and expressed his willingness to continue cooperating with follow-up treatment and to publish the case.

## Abbreviations

ALK, Anaplastic lymphoma kinase; CT, Computed tomography; EGFR, Epidermal growth factor receptor; EML4, Echinoderm microtubule-associated protein-like 4; MPR, Major pathological response; MRD, Molecular residual disease; MRI, Magnetic resonance imaging; NGS, Next-generation sequencing; NSCLC, Non-small cell lung cancer; OS, Overall survival; pCR, Pathological complete response; PET-CT, Positron emission tomography-computed tomography; PFS, Progression-free survival; PR, Partial response; R(un), Uncertain resection; TKI, Tyrosine kinase inhibitor; TMB, Tumor mutational burden; TTD, Time to discontinuation.
